# 1,1′-Bis(diphenylphosphino)ferrocene Platinum(II) Complexes as a Route to Functionalized Multiporphyrin Systems

**DOI:** 10.3390/nano11010178

**Published:** 2021-01-13

**Authors:** Maria Rosaria Plutino, Andrea Romeo, Maria Angela Castriciano, Luigi Monsù Scolaro

**Affiliations:** 1 CNR-ISMN, Istituto per lo Studio dei Materiali Nanostrutturati c/o Dipartimento di Scienze Chimiche, Biologiche, Farmaceutiche ed Ambientali, University of Messina V.le F. Stagno D’Alcontres, 31, 98166 Messina, Italy; mariarosaria.plutino@cnr.it (M.R.P.); anromeo@unime.it (A.R.); lmonsu@unime.it (L.M.S.); 2 Dipartimento di Scienze Chimiche, Biologiche, Farmaceutiche ed Ambientali and C.I.R.C.M.S.B., University of Messina V.le F. Stagno D’Alcontres, 31, 98166 Messina, Italy

**Keywords:** porphyrins, platinum(II) complexes, ferrocene, spectroscopic investigation, self-assembly

## Abstract

In this study, the cationic complex [PtMe(Me_2_SO)(dppf)]CF_3_SO_3_ (PtFc) (dppf = 1,1′-bis(diphenylphosphino)ferrocene) was exploited as a precursor to functionalize the multi-chromophoric system *hexakis*(pyridyl-porphyrinato)benzene (1). The final adduct [PtFc]_18_-1, containing eighteen platinum(II) organometallic [PtMe(dppf)] fragments, was prepared and characterized through UV/Vis absorption, ^31^P{^1^H}-NMR spectroscopy, and fluorescence emission. UV/vis and fluorescence titrations confirmed the coordination between the platinum(II) center and all the pyridyl moieties of the peripheral substituent groups of the porphyrin. The drop casting of diluted dichloromethane solution of [PtFc]_18_-1 onto a glass surface afford micrometer-sized emissive porphyrin rings.

## 1. Introduction

**Scheme 1:**
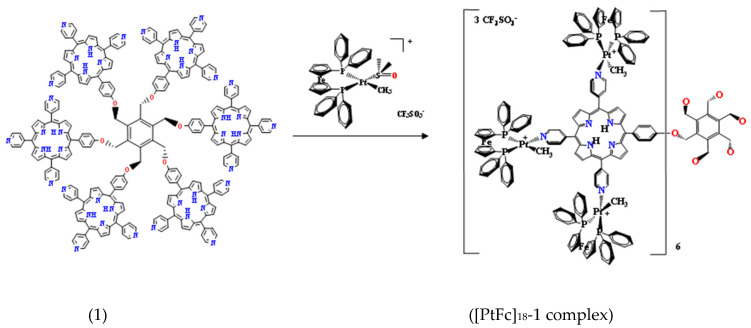
Synthetic route to the [PtFc]_18_-1 complex by a direct reaction of the porphyrin hexamer 1 and the platinum precursor complex PtFc.

Porphyrins and their metal complexes due to peculiar structural, electronic, and catalytic features are excellent molecular building blocks for accessing supramolecular systems for application in many fields, from energy storage and conversion to supramolecular catalysis, optics, and electronics [1,2,3,4,5]. Most of these properties can be properly tuned by a careful choice of the peripheral substituent groups, by the coordinated metal ion, and by their aggregation state. Metal-mediated self-assembly is an interesting approach for accessing well-defined porphyrin structures [6,7,8,9,10,11,12,13]. The shape and size of the supramolecular architectures are strictly related to the geometry imposed by the metal ions coordinated either in the macrocycle core or in its periphery and, in addition, by the number and relative orientations of the porphyrin substituent groups. In this framework, porphyrins bearing peripheral platinum(II) complexes have been reported and exhibit peculiar steric and electronic properties [14]. Some of these species have shown the annihilation of tumor cells combining the anticancer activity of the platinum complexes and the photo-physical behavior of the porphyrins for photodynamic therapy (PDT) [15,16,17,18,19]. Other examples have demonstrated the selective and controllable photoactivation of drugs within a tumor, reducing the adverse effects [20]. Clover-like shape porphyrin-bridged tetranuclear platinum(II) complexes have been reported for their ability to stabilize various kinds of G-quadruplexes, rather than the double-stranded DNA structure [16]. In the past, we have reported the use of platinum(II) organometallic complexes of the type [PtMe(Me_2_SO)(N–N)]^+^, where N–N is a series of diamines or diimines, as versatile building blocks for functionalizing the symmetric meso-tetrakis(4-pyridil)porphine (TpyP) [21,22,23,24,25]. Kinetic studies on these cationic platinum(II) complexes revealed that the exchange rate of the coordinated dimethylsulfoxide depends on the nature of the chelating ligand and is very labile when N-N is a diimine [26]. Complexes with different diimines bearing various substituent groups allowed tetra-substituted porphyrins soluble in micellar phases [21] and able to self-organize into mesoscopic structures to be obtained [22]. This methodology has been extended to organometallic platinum(II) complexes containing ligands able to perform other functions, and in particular, able to be redox active, such as in the complex ion [Pt(dppf)Me(Me_2_SO)]^+^ (dppf = 1,1′-bis(diphenylphosphino)ferrocene) [23]. Platinum(II) complexes containing dppf are multipurpose compounds and have applications in many research fields [27,28,29]. The majority of the reported complexes are mononuclear, with a single or two chelating dppf ligands, although *trans* coordination with a weak dative bond with iron has also been reported [30,31,32,33,34,35,36,37,38,39,40,41,42,43]. This versatile ligand adopts various modes of coordination and due to its large *trans*-labilizing ability with respect to a diimine [44], has been used as a corner joint for accessing platinum complexes’ molecular boxes [45]. In order to increase the π–stacking surface and obtain better-defined structures, a porphyrin hexamer was synthesized by coupling six pyridyl substituted porphyrin moieties to a central benzene core via an ether linkage (see compound 1 in Scheme 1) [46]. Further extension of the surface has been achieved through the metal-directed self-assembly of platinum diimine complexes, and the formation of mesoscopic assemblies on the surface has been reported [46]. In this case, despite the presence of eighteen potential coordination sites, the final multi-chromophoric superstructures only showed the coordination of six platinum complexes with the pyridine groups in the periphery of the hexameric porphyrin [46]. Here, we report the functionalization of this porphyrin hexamer by appending organometallic platinum(II) complexes containing the dppf ligand (see the [PtFc]_18_-1 complex in Scheme 1).

The introduction of ferrocenyl fragments on porphyrin rings is a topic of relevant interest, particularly in terms of their donor–acceptor complementary and electrochemical activities [47]. Several strategies have been reported in the literature for achieving functionalized porphyrin with ferrocene fragments, in order to investigate several processes, such as photoinduced electron transfer [48,49] and multi-electron redox catalysis [50], and to develop molecular-based electronic devices [51,52,53,54] or molecular electrogenic sensors [55]. We anticipate that the coordination of eighteen platinum(II) moieties with the pyridine groups can be achieved through a very simple synthetic route. The spectroscopic characterization of the final compound by means of NMR, absorption, and fluorescence emission spectroscopy is described in this paper. Moreover, as for similar systems previously reported, well-defined emissive nanostructured rings are easily achieved by the simple evaporation of CH_2_Cl_2_ solutions on a glass surface.

## 2. Materials and Methods

Chemicals. Porphyrin hexamer (1) and [PtMe(Me_2_SO)(dppf)]CF_3_SO_3_ (PtFc) were synthesized as previously reported in the literature [23,46]. The identity and purity of the samples were established by NMR spectroscopy. All solvents were of a reagent grade, and were available from commercial suppliers (Analytical Reagent Grade, Lab-Scan Ltd., Dublin, Ireland).

Synthesis. A total of 2 mg of hexamer 1 (0.000506 mmol) was added to a solution obtained by dissolving 9 mg of [PtMe(Me_2_SO)(dppf)]CF_3_SO_3_ (0.00911 mmol; [PtFc]/[1] = 18) in 30 mL of dichloromethane. The reaction mixture was exposed to stirring at room temperature for ca. 12–15 h in the dark. The solution was then completely evaporated under reduced pressure, washed several times with methanol to remove the excess dimethylsulfoxide, dissolved in the minimum volume of dichloromethane, and added to *n*-hexane until complete precipitation of the dark-red solid. The mixture was kept in a freezer at −35 °C for 3 h and the solvent was then removed to obtain the final compound [PtFc]_18_-1 with an 85% yield.

Nuclear Magnetic Resonance (NMR)

^31^P{^1^H} NMR (CD_2_Cl_2_): δ 29.5 (m,br, ^1^J_PtPA_ = 1968 Hz, *P*_A_ trans to CH_3_), 14.6 (m, br, ^1^J_PtPB_ = 3970 Hz, *P*_B_ trans to py).

Spectroscopy UV/Vis

UV/Vis (CH_2_Cl_2_) [λ_abs/_nm 430 (B-band), 522, 560, 600, 650 nm (Q-bands)]: Fluorescence emission (CH_2_Cl_2_) [λ_abs/_nm 660, 722].

### Instrumentation

NMR spectra were measured on a Bruker AMX R-300 spectrometer (Milan, Italy) equipped with a broad-band probe operating at 300.13 and 121.5 MHz for ^1^H and ^31^P nuclei, respectively. NMR chemical shifts are reported in parts per million (δ/ppm), and coupling constants are given in Hertz (J/Hz). All spectra were recorded in CD_2_Cl_2_ at 298 K. ^1^H NMR peaks were reported relative to tetramethylsilane (TMS), and referenced using the residual solvent resonances. ^31^P{^1^H} NMR chemical shifts are reported relative to external H_3_PO_4_.

UV/Vis absorption spectra were acquired on a Agilent mod. HP 8453 diode array spectrophotometer (Milan, Italy) using 1 cm path-length quartz cells. Fluorescence emission spectra were recorded on a Jasco mod. FP-750 spectrofluorimeter (Lecco, Italy).

Glass microscope slides were used as substrates for depositing samples. They were cleaned prior to use by immersion in a 1:1 mixture of concentrated NH_3_ solution and 30% H_2_O_2_ (Caution! This mixture is highly corrosive and should be handled with care under a ventilated fume-hood, avoiding skin contact), and then rinsed with Millipore-Q water and dried under an N_2_ stream. In total, 25 μL of sample solutions was cast onto the slides and evaporated at room temperature under a gentle N_2_ stream. Images were obtained by using a Zeiss Axiovert S100 optical microscope (Milan, Italy), working in fluorescence and transmission acquisition modes, with a CARV confocal module. The microscope was equipped with Plan Neofluar 10×/n.a.0.3 and 50×/n.a.0.5 (long working distance) objectives (Milan, Italy). A 100 W Hg lamp was used for fluorescence imaging.

## 3. Results and Discussion

The strategy previously used to obtain a tetranuclear platinum(II) complex containing *tetrakis*(4-pyridyl)porphyrin as the central core [21,22,23,24,46] was exploited to functionalize the *hexakis*(pyridyl-porphyrinato)benzene (1) according to Scheme 1. The precursor platinum(II) complex [PtMe(Me_2_SO)(dppf)]CF_3_SO_3_ was prepared in an almost quantitative yield by reacting *trans*-[PtMeCl(Me_2_SO)_2_] and the diphosphane ligand dppf in dichloromethane at 298 K and extracting the chloride by silver triflate according to a literature method [23]. The multi-porphyrin system 1, synthesized according to a procedure already reported in the literature [46], is characterized by the presence of eighteen potential coordination sites for the Pt(II), i.e., three pyridyl groups present in the *meso* position of each porphyrin unit linked to the central core.

**Figure 1:**
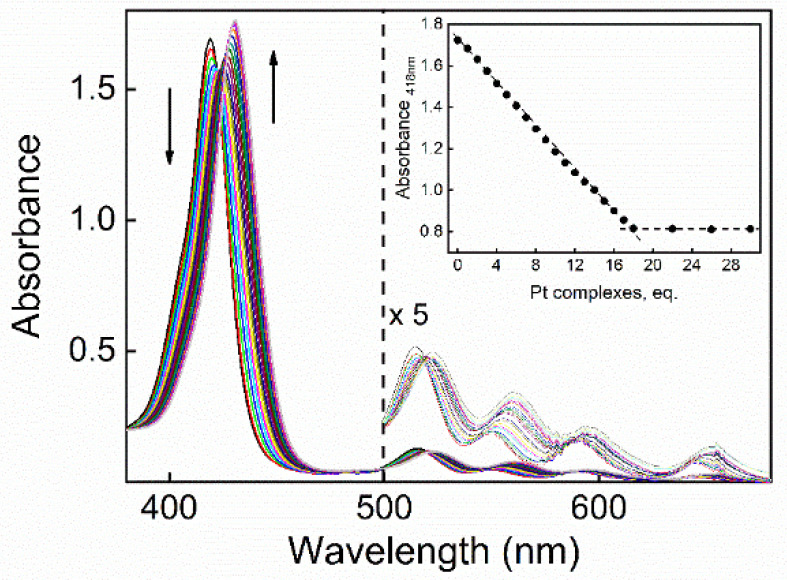
UV/Vis spectral changes upon the addition of PtFc to a 3 μM solution of porphyrin hexamer 1 in dichloromethane. Each spectrum corresponds to the addition of one equivalent of the metal complex. The inset shows the UV/Vis titration at λ 418 nm.

The gradual coordination of platinum(II) fragments with the pyridyl groups of 1 can be easily monitored in situ by UV/Vis and fluorescence emission spectroscopy, by titrating a solution of 1 with known aliquots of a solution of the PtFc complex. Upon the addition of this latter component, the electronic spectra of the multi-porphyrin systems show the progressive decrease of the initial B-band centered at 418 nm and a matching increase of a new band bathochromically shifted to 430 nm (Figure 1). The absence of a clear isosbestic point suggests the presence of intermediate species due to partial coordination of the available sites. A UV/Vis titration, measuring the absorbance at 418 nm, is displayed in the inset of Figure 1. A clear break point corresponding to eighteen Pt equivalents is evident. The coordination of the metal complexes at all of the available sites is a rather interesting result. Indeed, a previous investigation reported that Pt(II) complexes bearing diimine ligands with long alkyl chains are only able to bind six sites in the periphery of the hexameric structure [46]. The actual results can be explained as an interplay of (i) the steric hindrance of the bulky ferrocenyl groups of the dppf ligand and (ii) the higher lability of Me_2_SO as a consequence of the stronger *trans* influence and *trans* effect of a phosphane with respect to nitrogen ligands, which confer a higher reactivity to the metal complex PtFc with respect to analogous diimine containing platinum(II) complexes [46]. In the case of these latter metal precursors, coordination of the metal fragments exclusively occurs on pyridyl groups in a *trans* position with respect to the ether linkage. Even if introducing a certain degree of steric hindrance in the periphery of each porphyrin unit, the involvement of only six outer pyridyl moieties does not prevent stacking interactions between adjacent porphyrin rings. Indeed, the occurrence of three stacked pairs was confirmed by molecular modeling and NMR studies, even in the porphyrin hexamer 1 that was not complexed [46,56]. In the case of [PtFc]_18_-1, the invariance of the shape of the UV/Vis profile after the coordination of the platinum fragments suggests the absence of perturbation in the aromatic systems and charge transfer between the ferrocene and the porphyrin subunits [57]. The binding of [PtMe(dppf)] moieties to all of the available pyridyl sites completely removes any possibility for stacking interactions among porphyrin units in the supramolecular structure.

**Figure 2:**
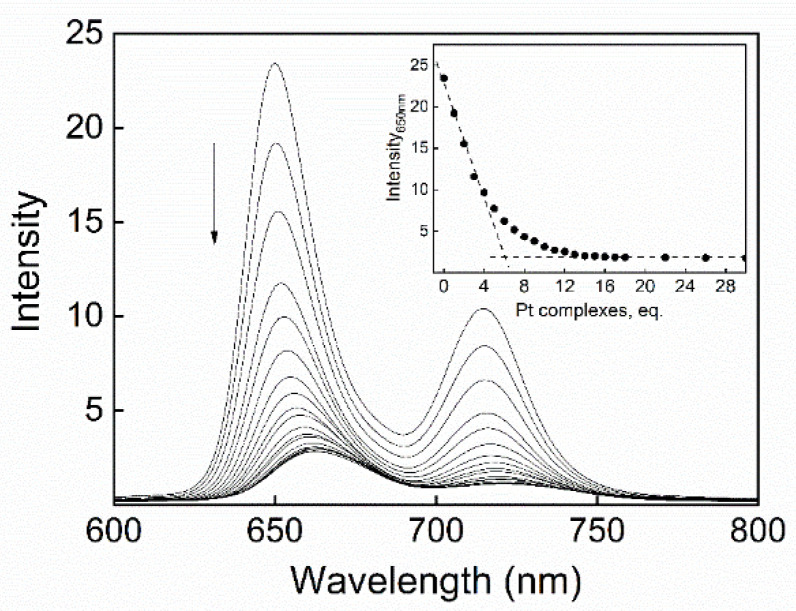
Fluorescence emission spectral changes upon the addition of PtFc to a 3 μM solution of hexamer **1** in dichloromethane. Each spectrum corresponds to the addition of one equivalent of the metal complex. The inset shows the fluorescence emission titration (λ_em_ = 650 nm; λ_ex_ = 424 nm).

The fluorescence emission spectra exhibit a gradual and partial quenching in their intensity, together with a bathochromic shift (∆λ = + 12 nm) of the bands typical of **1**, upon the addition of PtFc complexes (Figure 2). The titration curve reported in the inset of Figure 2 shows a gradual decrease in the intensity, which stabilizes after the addition of six PtFc equivalents. These data suggest that the coordination of only one Pt(II) fragment on each porphyrin is enough to induce a substantial quenching of the fluorescence emission. This apparent discrepancy between the two techniques is probably due to the heavy atom effect induced by coordination of the metal ions with the chromophores. This effect affords a spin-orbit coupling perturbation, influencing the spin-selection rules for radiative and radiationless transitions. In a simple molecular system, quenching of the lowest excited singlet state (S_1_) in the presence of a heavy atom is expected, as a consequence of enhanced intersystem crossing (S_1_/T_1_) [58]. It has been reported in the literature that for porphyrins bearing pyridine groups, the coordination with platinum(II) decreases the intersystem crossing quantum yield. The internal conversion process is also influenced when changing the position of the metal fragment on the pyridine substituent. Moreover, the magnitude and shape of the singlet and triplet excited state absorption cross section are affected by the charge redistribution due to the presence of platinum(II) [14].

**Figure 3:**
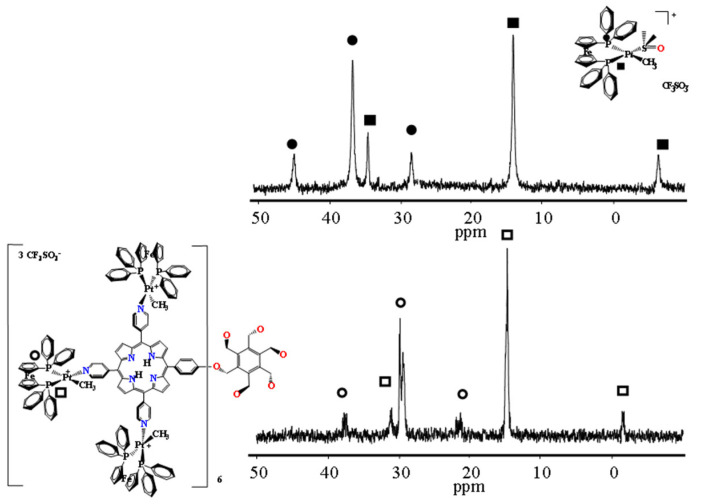
^31^P{^1^H} NMR spectra of the PtFc precursor (upper) and fully platinated [PtFc]_18_-1 in CD_2_Cl_2_, where T = 298 K.

In order to isolate the pure compound, we carried out the reaction between the hexamer **1** and the precursor PtFc at a 1:18 stoichiometric ratio in dichloromethane solution. After the reaction, the excess dimethylsulfoxide was removed by washing the sample with methanol and precipitation in a low polar solvent, thus producing a pure compound in about an 85% yield. The complete coordination of all Pt (II) complexes on all of the available pyridine groups was confirmed by the ^31^P{^1^H} NMR spectroscopy. The ^31^P{^1^H} NMR spectra reported in Figure 3 show a comparison of the starting building block PtFc and the final adduct [PtFc]_18_-1. An upfield shift of the peak relative to phosphorous in a *trans* position with respect to the methyl group from δ 36.2 to 29.5 ppm is evident. The value of ^1^J_Pt-P_ = 1968 Hz is in line with the high *trans* influence of the methyl group. On the contrary, the peak relative to phosphorous in a *trans* position with respect to the pyridyl group undergoes a slight downfield shift (∆δ = +0.7 ppm), while a consistent change can be observed in the value of the ^1^J_Pt-P_ from 4919 (PtFc complex) to 3970 Hz ([PtFc]_18_-1). This reduction is in agreement with the higher *trans*-influence of the dmso ligand with respect to pyridine [59]. Analogous to other multi-porphyrin systems [56], the ^1^H NMR signals are rather broad and uninform, probably due to fast exchange occurring between different conformers at room temperature; the slow tumbling motion of the final platinated [PtFc]_18_-1 large molecule also affects the proton resonance line widths by causing long relaxation times and undesired broadening phenomena.

**Figure 4:**
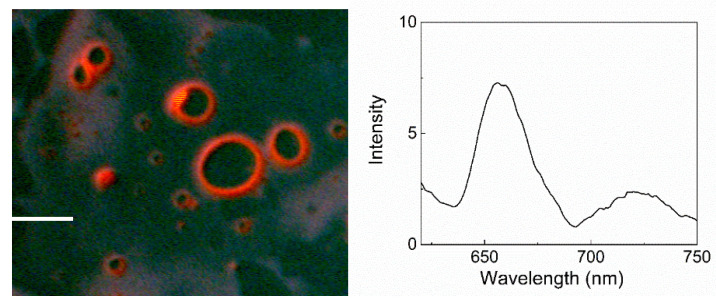
Fluorescence confocal optical image of samples obtained by the drop casting of a dichloromethane solution of [PtFc]_18_-1 on a glass cover slide (left) and the corresponding fluorescence emission spectrum (right) (λ_ex_ = 424 nm; the bar is 5 μm).

Deposition of the final compound on glass cover slides was achieved by direct drop casting of a micromolar dichloromethane solution. Optical microscopy images of the samples show the formation of rings comparable to those already reported for similar systems [46,56], whose diameters span from 0.5 to 10 μm and thicknesses range from about 100 to 500 nm. The rings are still emissive in the solid state, as revealed by fluorescence confocal optical microscopy (Figure 4). The emission spectra, analogous to the solution phase, show the two-band profile, with maxima at 660 and 720 nm for Q (0,0) and Q (0,1) transitions, respectively. The invariance of the spectroscopic behavior in the solution and in a solid state suggests the absence, in the latter, of π–interactions between the porphyrin planes, probably due to the presence of multiple charges of the platinum complexes. Nevertheless, the interesting formation of well-defined structures can be explained in terms of a balance between physical dewetting processes and porphyrin aggregation behavior driven by directional supramolecular interactions. The growth of well-defined patterns of porphyrins on different solid substrates has been well-reported [23,46,56,60], but to the best of our knowledge, this is the first example of a porphyrin supramolecular structure coordinated with such a high number of platinum complexes bearing ferrocene ligands.

## 4. Conclusions

Functionalization of a large chromophoric system can usually be achieved through complicated synthetic steps. Here, we describe a facile route for obtaining a highly substituted porphyrin hexamer bearing metal fragments with redox active groups. Even if the fluorescence emission of the multi-porphyrin system is partially quenched by the presence of heavy atoms in the peripheral substituent groups, this property is retained, even for a solid phase. Dewetting phenomena, similar to porphyrin systems, lead to the formation of well-defined rings. These micrometer-sized structures, together with interesting photophysical properties and the presence of redox active centers, whose behavior should be studied in future investigations, offer a way of exploring potential applications in nanocatalysis or optoelectronics.

## Data Availability

No applicable.

## References

[1] Drain C.M., Varotto A., Radivojevic I. (2009). Self-Organized Porphyrinic Materials. Chem. Rev..

[2] Lutzen A., Muller S.C., Parisi J. (2015). Supramolecular Organization of pi-Conjugated Oligomers. Bottom-Up Self-Organization in Supramolecular Soft Matter: Principles and Prototypical Examples of Recent Advances.

[3] Elemans J., Van Hameren R., Nolte R.J.M., Rowan A.E. (2006). Molecular materials by self-assembly of porphyrins, phthalocyanines, and perylenes. Adv. Mater..

[4] Hoeben F.J.M., Jonkheijm P., Meijer E.W., Schenning A. (2005). About supramolecular assemblies of pi-conjugated systems. Chem. Rev..

[5] Milic T.N., Chi N., Yablon D.G., Flynn G.W., Batteas J.D., Drain C.M. (2002). Controlled hierarchical self-assembly and deposition of nanoscale photonic materials. Angew. Chem. Int. Ed..

[6] Gianferrara T., Serli B., Zangrando E., Iengo E., Alessio E. (2005). Pyridylporphyrins peripherally coordinated to ruthenium-nitrosyls, including the water-soluble Na-4 Zn center dot 4′ TPyP{RuCl_4_(NO)}_4_: Synthesis and structural characterization. New J. Chem..

[7] Iengo E., Zangrando E., Minatel R., Alessio E. (2002). Metallacycles of porphyrins as building blocks in the construction of higher order assemblies through axial coordination of bridging ligands: Solution- and solid-state characterization of molecular sandwiches and molecular wires. J. Am. Chem. Soc..

[8] Iengo E., Milani B., Zangrando E., Geremia S., Alessio E. (2000). Novel Ruthenium Building Blocks for the Efficient Modular Construction of Heterobimetallic Molecular Squares of Porphyrins. Angew. Chem. Int. Ed..

[9] Alessio E., Ciani E., Iengo E., Kukushkin V.Y., Marzilli L.G. (2000). Stepwise assembly of unsymmetrical supramolecular arrays containing porphyrins and coordination compounds. Inorg. Chem..

[10] Prodi A., Indelli M.T., Kleverlaan C.J., Scandola F., Alessio E., Gianferrara T., Marzilli L.G. (1999). Side-to-face ruthenium porphyrin arrays: Photophysical behavior of dimeric and pentameric systems. Chem. Eur. J..

[11] Naik A., Rubbiani R., Gasser G., Spingler B. (2014). Visible-Light-Induced Annihilation of Tumor Cells with Platinum-Porphyrin Conjugates. Angew. Chem. Int. Ed..

[12] Maran U., Britt D., Fox C.B., Harris J.M., Orendt A.M., Conley H., Davis R., Hlady V., Stang P.J. (2009). Self-Assembly of a Triangle-Shaped, Hexaplatinum-Incorporated, Supramolecular Amphiphile in Solution and at Interfaces. Chem. Eur. J..

[13] Suijkerbuijk B., Gebbink R. (2008). Merging porphyrins with organometallics: Synthesis and applications. Angew. Chem. Int. Ed..

[14] Cocca L.H.Z., Gotardo F., Sciuti L.F., Acunha T.V., Iglesias B.A., de Boni L. (2018). Investigation of excited singlet state absorption and intersystem crossing mechanism of isomeric meso-tetra(pyridyl)porphyrins containing peripheral polypyridyl platinum(II) complexes. Chem. Phys. Lett..

[15] Tong K.-C., Hu D., Wan P.-K., Lok C.-N., Che C.-M., Sadler P.J., van Eldik R. (2020). Chapter Three—Anti-cancer gold, platinum and iridium compounds with porphyrin and/or N-heterocyclic carbene ligand(s). Advances in Inorganic Chemistry.

[16] Cao Q., Cao Q., Ding Y.L., Zhong Y.F., Mu G., Qin P.Z., Ji L.N., Mao Z.W. (2015). Platinum(II) clovers targeting G-quadruplexes and their anticancer activities. Dalton Trans..

[17] Yao S., Chen L., Jia F., Sun X., Su H., Liu H., Yang L., Wang Z., Wu F., Wang K. (2019). Platinated porphyrin tailed with folic acid conjugate for cell-targeted photodynamic activity. J. Lumin..

[18] Volostnykh M.V., Borisov S.M., Konovalov M.A., Sinelshchikova A.A., Gorbunova Y.G., Tsivadze A.Y., Meyer M., Stern C., Bessmertnykh-Lemeune A. (2019). Platinum(II) and palladium(II) complexes with electron-deficient meso-diethoxyphosphorylporphyrins: Synthesis, structure and tuning of photophysical properties by varying peripheral substituents. Dalton Trans..

[19] Hu X., Ogawa K., Li S., Kiwada T., Odani A. (2019). A Platinum Functional Porphyrin Conjugate: An Excellent Cancer Killer for Photodynamic Therapy. Bull. Chem. Soc. Jpn..

[20] Wang Z., Wang N., Cheng S.-C., Xu K., Deng Z., Chen S., Xu Z., Xie K., Tse M.-K., Shi P. (2019). Phorbiplatin, a Highly Potent Pt(IV) Antitumor Prodrug That Can Be Controllably Activated by Red Light. Chem.

[21] Scolaro L.M., Donato C., Castriciano M., Romeo A., Romeo R. (2000). Micellar aggregates of platinum(II) complexes containing porphyrins. Inorg. Chim. Acta.

[22] Castriciano M., Romeo A., Romeo R., Scolaro L.M. (2002). Mesoscopic globular self-assemblies of platinum(II) complexes containing porphyrins. Eur. J. Inorg. Chem..

[23] Scolaro L.M., Plutino M.R., Romeo A., Romeo R., Ricciardi G., Belviso S., Albinati A. (2006). Platinum(II) complexes bearing 1,1′-bis(diphenylphosphino)ferrocene as building blocks for functionalized redox active porphyrins. Dalton Trans..

[24] Plutino M.R., Castriciano M.A., Mazzaglia A., Saporita M., Romeo A., Scolaro L.M. (2011). Synthesis and aggregation behavior of a novel water-soluble porphyrin platinum(II) terpyridine complex. J. Porphyr. Phthalocyanines.

[25] Chaves O.A., Acunha T.V., Iglesias B.A., Jesus C.S.H., Serpa C. (2020). Effect of peripheral platinum(II) bipyridyl complexes on the interaction of tetra-cationic porphyrins with human serum albumin. J. Mol. Liq..

[26] Romeo R., Scolaro L.M., Nastasi N., Arena G. (1996). Rates of dimethyl sulfoxide exchange in monoalkyl cationic platinum(II) complexes containing nitrogen bidentate ligands. A proton NMR study. Inorg. Chem..

[27] Štĕpnička P. (2008). Ferrocenes: Ligands, Materials and Biomolecules.

[28] Teo P., Koh L.L., Andy Hor T.S. (2008). Spacer-Directed Coordination Polymers-of-Oligomers (POLO) of Silver. Inorg. Chem..

[29] Togni A., Hayashi T. (1995). Ferrocenes: Homogeneous Catalysis, Organic Synthesis, Materials Science.

[30] Ares R., López-Torres M., Fernández A., Pereira M.T., Alberdi G., Vázquez-García D., Fernández J.J., Vila J.M. (2003). Functionalized cyclopalladated compounds with bidentate Group 15 donor atom ligands: The crystal and molecular structures of [{Pd[5 -(COH)C_6_H_3_C(H)NCy-C_2_,N](Cl)}2(μ-Ph_2_PRPPh_2_)] (R=CH_2_CH_2_, Fe(C_5_H_4_)_2_], [Pd{5-(COH)C_6_H_3_C(H)NCy-C_2_,N}(Ph_2_PCH_2_PPh_2_-P,P)][PF_6_] and [Pd{5-(COH)C_6_H_3_C(H)N(Cy)-C_2_,N}(Ph_2_PCH_2_CH_2_AsPh_2_-P,As)][PF_6_]. J. Organomet. Chem..

[31] Evans R.C., Douglas P., Winscom C.J. (2006). Coordination complexes exhibiting room-temperature phosphorescence: Evaluation of their suitability as triplet emitters in organic light emitting diodes. Coord. Chem. Rev..

[32] Jamali S., Nabavizadeh S.M., Rashidi M. (2008). Binuclear Cyclometalated Organoplatinum Complexes Containing 1,1′-Bis(diphenylphosphino)ferrocene as Spacer Ligand: Kinetics and Mechanism of MeI Oxidative Addition. Inorg. Chem..

[33] Lu W., Mi B.X., Chan M.C.W., Hui Z., Che C.M., Zhu N., Lee S.T. (2004). Light-Emitting Tridentate Cyclometalated Platinum(II) Complexes Containing σ-Alkynyl Auxiliaries:  Tuning of Photo- and Electrophosphorescence. J. Am. Chem. Soc..

[34] Ma J.F., Yamamoto Y. (2000). Reaction of di-μ-dichloro-bis(N,N-dimethylbenzylamine-C2,N)dipalladium(II) with diphosphines. Six-membered ring complexes bearing spiro rings. Inorg. Chim. Acta.

[35] Ravindranathan D., Vezzu D.A.K., Bartolotti L., Boyle P.D., Huo S. (2010). Improvement in Phosphorescence Efficiency through Tuning of Coordination Geometry of Tridentate Cyclometalated Platinum(II) Complexes. Inorg. Chem..

[36] Sato M., Shigeta H., Sekino M., Akabori S. (1993). Synthesis, some reactions, and molecular structure of the Pd(BF4)2 complex of 1,1′-bis(diphenylphosphino)ferrocene. J. Organomet. Chem..

[37] Smith D.C., Haar C.M., Stevens E.D., Nolan S.P. (2000). Synthetic, Structural, and Solution Calorimetric Studies of Pt(CH_3_)_2_(PP) Complexes. Organometallics.

[38] Sun W., Zhu H., Barron P.M. (2006). Binuclear Cyclometalated Platinum(II) 4,6-Diphenyl-2,2′-bipyridine Complexes:  Interesting Photoluminescent and Optical Limiting Materials. Chem. Mater..

[39] Thorn D.L., Fultz W.C. (1989). Rhodamine complexes. Preparation, and photophysical properties, and the structure of [Rh(rhodamine)(CO)(P(tol)_3_)_2_][SbF_6_]. J. Phys. Chem..

[40] Wilson J.J., Fedoce Lopes J., Lippard S.J. (2010). Synthesis, Characterization, and Photophysical Properties of Three Platinum(II) Complexes Bearing Fluorescent Analogues of the Di-2-pyridylmethane Ligand. Inorg. Chem..

[41] Wong W.Y., He Z., So S.K., Tong K.L., Lin Z. (2005). A Multifunctional Platinum-Based Triplet Emitter for OLED Applications. Organometallic.

[42] Bandoli G., Dolmella A. (2000). Ligating ability of 1,1′-bis(diphenylphosphino)ferrocene: A structural survey (1994–1998). Coord. Chem. Rev..

[43] Shahsavari H.R., Rashidi M., Nabavizadeh S.M., Habibzadeh S., Heinemann F.W. (2009). A Tetramethylplatinum(IV) Complex with 1,1′-Bis(diphenylphosphanyl)ferrocene Ligands: Reaction with Trifluoroacetic Acid. Eur. J. Inorg. Chem..

[44] Appleton T.G., Clark H.C., Manzer L.E. (1973). Trans-influence—Its measurement and significance. Coord. Chem. Rev..

[45] Leininger S., Olenyuk B., Stang P.J. (2000). Self-assembly of discrete cyclic nanostructures mediated by transition metals. Chem. Rev..

[46] Lensen M.C., Castriciano M., Coumans R.G.E., Foekema J., Rowan A.E., Scolaro L.M., Nolte R.J.M. (2002). Hexakis (pyridyl-functionalised porphyrinato)benzene as a building block for the construction of multi-chromophoric arrays. Tetrahedron Lett..

[47] Bucher C., Devillers C.H., Moutet J.C., Royal G., Saint-Aman E. (2009). Ferrocene-appended porphyrins: Syntheses and properties. Coord. Chem. Rev..

[48] Gust D., Moore T.A., Moore A.L. (1993). Molecular mimicry of photosynthetic energy and electron transfer. Acc. Chem. Res..

[49] Wasielewski M.R. (1992). Photoinduced electron transfer in supramolecular systems for artificial photosynthesis. Chem. Rev..

[50] Bard A.J. (1995). Electron transfer branches out. Nature.

[51] Heath J.R., Kuekes P.J., Snider G.S., Williams R.S. (1998). A Defect-Tolerant Computer Architecture: Opportunities for Nanotechnology. Science.

[52] Chen Y., Jung G.-Y., Ohlberg D.A.A., Li X., Stewart D.R., Jeppesen J.O., Nielsen K.A., Stoddart J.F., Williams R.S. (2003). Nanoscale molecular-switch crossbar circuits. Nanotechnology.

[53] Liu Z., Yasseri A.A., Lindsey J.S., Bocian D.F. (2003). Molecular Memories that Survive Silicon Device Processing and Real-World Operation. Science.

[54] Matsushige K., Yamada H., Tada H., Horiuchi T., Chen X.Q. (1998). Nanoscopic Molecular Memories. Ann. N. Y. Acad. Sci..

[55] Beer P.D., Gale P.A., Chen G.Z. (1999). Mechanisms of electrochemical recognition of cations, anions and neutral guest species by redox-active receptor molecules. Coord. Chem. Rev..

[56] Biemans H.A.M., Rowan A.E., Verhoeven A., Vanoppen P., Latterini L., Foekema J., Schenning A., Meijer E.W., de Schryver F.C., Nolte R.J.M. (1998). Hexakis porphyrinato benzenes. A new class of porphyrin arrays. J. Am. Chem. Soc..

[57] Sohn Y.S., Hendrickson D.N., Gray H.B. (1971). Electronic structure of metallocenes. J. Am. Chem. Soc..

[58] Prodi A., Kleverlaan C.J., Indelli M.T., Scandola F., Alessio E., Iengo E. (2001). Photophysics of Pyridylporphyrin Ru(II) Adducts: Heavy-Atom Effects and Intramolecular Decay Pathways. Inorg. Chem..

[59] Pregosin P.S., Webb G.A. (1986). Platinum NMR Spectroscopy. Annual Reports on NMR Spectroscopy.

[60] van Hameren R., van Buul A.M., Castriciano M.A., Villari V., Micali N., Schon P., Speller S., Scolaro L.M., Rowan A.E., Elemans J.A.A.W. (2008). Supramolecular porphyrin polymers in solution and at the solid-liquid interface. Nano Lett..

